# M6A-Related Bioinformatics Analysis Reveals a New Prognostic Risk Signature in Cutaneous Malignant Melanoma

**DOI:** 10.1155/2022/8114731

**Published:** 2022-06-06

**Authors:** Qingxiong Yu, Hainan Zhu, Huijing Wang, Rehanguli Aimaier, Manhon Chung, Zhichao Wang, Qingfeng Li

**Affiliations:** ^1^Department of Plastic and Reconstructive Surgery, Shanghai Ninth People's Hospital, Shanghai Jiao Tong University School of Medicine, 639 Zhizaoju Road, Shanghai 200011, China; ^2^Department of Plastic Surgery, The Second Affiliated Hospital Zhejiang University School of Medicine, Hangzhou 310017, China

## Abstract

Cutaneous malignant melanoma (CMM) is the most deadly skin cancer worldwide. Despite advances in the treatments of CMM, its incidence and mortality rates are still increasing. N6-methyladenosine (m6A) is the most common form of RNA modification and has attracted increasing interest in cancer initiation and progression. However, the role of m6A regulators in CMM and their correlation with prognosis remain elusive. Here, we demonstrated that by applying consensus clustering, all CMM patient cases can be divided into two clusters based on overall expression levels of 25 m6A genes. We systematically analyzed the prognostic value of the 25 m6A RNA methylation regulators in CMM and found that ELAVL1, ABCF1, and IGF2BP1 yield the highest scores for predicting the prognosis of CMM. Accordingly, we derived a risk signature consisting of three selected m6A genes as an independent prognostic marker for CMM and validated our findings with data derived from a different CMM cohort. Next, we determined that CNVs in m6A genes had a significant negative impact on patient survival. The mRNA expression levels of m6A genes were correlated with CNV mutation. Moreover, in the selected three risk signature m6A regulators, GSEA analysis showed that they were closely correlated with inflammation and immune pathways. TME analysis proved that m6A gene expressions were negatively correlated with immune cell infiltration. In conclusion, m6A regulators are vital participants in CMM pathology; and ELAVL1, ABCF1, and IGF2BP1 mRNA levels are valuable factors for prognosis prediction and treatment strategy development.

## 1. Introduction

Malignant melanoma is a highly malignant tumor derived from melanocytes. It mostly occurs in the skin, digestive tract, uvea oculi, pia mater, genitalia, and nasal cavity. Among them, cutaneous malignant melanoma (CMM) is the most popular subtype [1]. CMM has the characteristics of high malignancy, high invasion, easy metastasis, and poor prognosis. Although CMM accounts for only 4% of all skin tumors, but it is more aggressive and fatal than other forms of skin cancer and accounting for 75% of skin cancer-related deaths [2]. In recent years, the incidence rate of CMM has increased year by year. It is estimated that 100,350 new melanoma cases (60,190 in men and 40,160 in women) are expected to be diagnosed in 2020 in the United States, and about 8% of these patients will be die of this disease [3]. To date, a lot of researches have been done in CMM, but the underlying molecular mechanisms still unclear [4, 5].

Biomarkers facilitate the diagnosis and identification of individualized characteristics of melanoma, which would benefit for precision therapy [6, 7]. Therefore, it is vital to identify potential molecular prognostic biomarkers in melanoma. Since epigenetic modification plays an important role in tumor development, more and more epigenetic biomarkers have been found in melanoma in recent years. DNA methylation is the most studied epigenetic biomarker in malignant melanoma [8]. In general, methylation in the promoter region inhibits the expression of genes. Methylation of tumor suppressor genes (PTEN, p16, RASSF1A, etc.) is usually found in malignant melanoma and is related to tumor progression. Wouters et al. found that the methylation PON3 genes can be used as a supplement to the classical markers of malignant melanoma [9]. Falzone et al. reported that the methylation of MMP9 gene leads to overexpression of MMP9 gene, which promotes the progression and metastasis of melanoma [10]. However, the epigenetic biomarkers in melanoma are still limited to a few studies and have not been popularized. Therefore, the prognostic role of epigenetic biomarkers in malignant melanoma is still worthy of expanded.

There are about 172 different kinds of RNA modifications commensurate with the latest version of MODOMICS, a database of RNA modifications [11]. In particular, N6-methyladenosine (m6A) is one of the most extensive and exuberant internal posttranscriptional modifications in all kinds of RNA, especially in messenger RNA (mRNA) [12–14]. M6A modification on RNA is abundant near the stop codon and 3′-untranslated region (3′-UTR) and translated near 5′-UTR in a cap-independent manner, thereby regulating RNA transcription, translation, and metabolism [15]. The formation and regulation of m6A are manipulated by a methyltransferase complex comprising three category proteins including “writers,” “erasers,” and “readers.” “writers” are methyltransferase catalyzing the formation of m6A, containing methyltransferase-like 3 (METTL3) as the core component and other related subunits including METTL14, WTAP, VIRMA, RBM15, and ZC3H13 [16]. “erasers” function as demethylases, including FTO and ALKBH5. “readers” are a group of RNA binding proteins that recognize the m6A methylation and perform corresponding functions. These proteins mainly include YT521-Bhomology (YTH) domain containing protein families (YTHDCs), YTH N6-methyl-adenosine RNA binding protein families (YTHDFs), insulin-like growth factor 2 mRNA-binding protein families (IGF2BPs), and heterogeneous nuclear ribonucleoprotein (HNRNP) protein families [17].

Researches demonstrated that m6A methylation in mRNA is significantly associated with tumor proliferation, migration, invasion, and metastasis during the process of cancer progression [14]. It is reported that FTO could modify the m6A level of MALAT and promotes bladder cancer progression [18]. METTL3-mediated m6A modification of ZBTB4 mRNA is involved in the smoking-induced EMT in lung cancer [19]. METTL14 inhibits the proliferation, migration, and invasion of gastric cancer by regulating the PI3K/AKT/mTOR signaling pathway [20]. WTAP facilitates progression of hepatocellular carcinoma via m6A-HuR-dependent epigenetic silencing of ETS1 [21]. The value of m6A methylation pattern on tumor microenvironment or prognosis was also demonstrated in gastric cancer and ovarian cancer [22, 23]. However, the role of m6A methylation in the development and progression of CMM remains questionable.

In this study, we aim to analyze the differentially expressing profiles of m6A-related genes in CMM subtype and establish a cox regression model to predict the overall survival. We systematically analyzed the expression of 25 central m6A regulators in 470 CMM with RNA sequencing data from The Cancer Genome Atlas (TCGA) datasets. We aimed to evaluate the values of m6A genes in predicting the prognosis of CMM patients and explore possible signaling pathways regulated by m6A regulators in CMM through comprehensive bioinformatics analyses. Based on LASSO and multivariate Cox regression models, we constructed a 3-gene (ELAVL1, ABCF1, and IGF2BP1) signature of m6A regulators with prognostic value in CMM that can effectively predict patient prognosis. Multivariate Cox regression analysis suggested that risk score might be an independent prognostic indicator for the patients with CMM. We further analyzed m6A gene mutation and expression profiles in CMM patients from TCGA database. We determined that the CNV in m6A regulatory genes had a significant negative impact on patient survival. The mRNA expression levels of gross m6A genes were significantly correlated with CNV mutation. We also conclude that high expression of m6A genes correlated with reduced immune cell infiltration in CMM, which may be responsible for poor prognosis.

## 2. Materials and Methods

### 2.1. Database

In October 2020, we obtained the RNA-seq transcriptome data and the corresponding clinicopathological information of 470 SKCM patients from the TCGA database (http://cancergenome.nih.gov/). For the RNA-seq data, TCGA samples were normalized by fragment per kilobase of exon model per million.

### 2.2. Clustering of CMM Patients by m6A RNA Methylation Regulator Expression

We collected a list of 25 m6A RNA methylation regulators from published literatures (Table S1). Next, we performed cluster analysis to analyze the 470 CMM samples from TCGA database (Ward's method) according to the m6A regulators' expression. Furthermore, clinical data and survival time were extracted from the CMM patients. Observed survival interval (OBS) was used in the survival analysis in this study [24]. The correlation analysis between clinical traits and clustering results was carry out in R software. Finally, the heatmap and survival chart were constructed by the ggplots package in R software. All data were processed using the R software (version 3.4.0).

### 2.3. M6A-Related Genes Obtained and Differential Analysis

Differentially expressed gene analysis was performed between metastatic and nonmetastatic cases in TCGA database. The *p* value was calculated by R deseq2 and edge package, respectively. The difference screening standard was *p* < 0.05, and fold change was more than 2 times. Either of the methods calculate *p* < 0.05 is considered to be significantly. GO analysis and KEGG enrichment based on these differentially expressed genes were done. Among them, m6A regulator genes of were screened out. Protein-protein interaction (PPI) analysis was conducted to reveal the molecular mechanisms of the 25 m6A RNA methylation regulators in CMM. We utilized the Search Tool for the Retrieval of Interacting Genes (STRING) protein database 11.0 (http://string-db.org/) to construct the PPI networks. An interaction score > 0.4 was regarded as the cut-off criterion. We further use UALCAN (http://ualcan.path.uab.edu/index.html) to analyze tumor subgroup gene expression and survival. Using TCGA transcriptome and clinical patient data, compare them across different tumor subgroups as defined by the patient's age and tumor grade through the expression level of the gene.

### 2.4. Bioinformatics Analysis

To evaluate the prognostic value of m6A genes, we executed univariate Cox regression analyses of their expression in the TCGA dataset, from which we selected six genes virtually associated with survival (*p* < 0.05), which we chose for further functional research and development of a potential risk signature with the LASSO Cox regression algorithm. The “forestplot” R packages were used to draw forest plots. Finally, three regulators and their coefficients were decided by the minimum criteria, choosing the best penalty parameter *λ* associated with the TGGA datasets. The risk formula was applied to count a risk score for each patient in TGGA datasets. The high-risk group (samples with the risk score higher than median score) and the low-risk subtype (samples with risk score lower than median score) were defined in CMM cases based on the risk score of its tumor samples. The data were processed by R packages “survival.” Kaplan-Meier curves were drawn to demonstrate the relationship between the patient's overall survival and gene expression levels of m6A RNA methylation regulators. The relationship was tested by the log-rank test. The ROC analysis was used for testing if the survival prediction is sensitive and specific based on the risk score.

### 2.5. Validation of the Risk Signature

To determine the robustness of this model, we tend to validate the same signature genes in the validation set. We search for melanoma cohort with gene expression datasets in GEO database; survival terms were also needed for survival analysis. The only eligible dataset (GSE65904) was downloaded and used for further validation. We used multivariate Cox proportional analysis to determine a panel of prognostic genes. The demographics and clinical information, including age and grade, were used for model correction. The calculation of the patient's risk score in the validation set was performed according to the formula obtained from the multivariate Cox proportional model.

### 2.6. Gene Set Enrichment Analysis (GSEA) of the Three Selected m6A Genes

Gene Set Enrichment Analysis (GSEA) of prognosis-related MeDEGs was performed using GSEA 3.0 software with gene set c2 (cp.kegg.v.6.2.symbols.gmt). High throughput RNA expression of 470 CMM genes from TCGA was utilized as the dataset. Each sample was defined as either “High expression” or “Low expression,” depending on whether it was greater than the median mRNA expression value of prognosis-related defined m6A regulators or not. The number and type of permutations were set at “1000” and “phenotype,” respectively. An enrichment score > 0.4 and *p* < 0.05 were regarded as statistically significant.

### 2.7. Mutation Analysis

CMM cases with CNV, mutation, and clinicopathological information were retrieved from the TCGA database. CNV was identified using segmentation analysis and GISTIC algorithm in the cBioportal platform. The relationship between clinicopathological characteristics was analyzed according to the status of CNV and/or mutation: “patients with mutation and/or CNV of m6A regulators” and “patients without CNV or mutation.” The patients were divided into two groups according to whether there was any mutation in the m6A genes. The single factor Cox regression analysis was performed to detect whether SNV, CNV, SNV, or CNV in the m6A gene are correlated with patient prognosis.

### 2.8. Estimation of TME Cell Infiltration

We used the ssGSEA (single-sample gene-set enrichment analysis) algorithm to quantify the relative abundance of each cell infiltration in the CMM TME. The gene set for marking each TME infiltration immune cell type was obtained from the study of Charoentong, which stored various human immune cell subtypes including activated CD8 T cell, activated dendritic cell, macrophage, natural killer T cell, and regulatory T cell. We further analyzed the correlation between m6A regulators and immune cell infiltration.

### 2.9. Statistical Analysis

One-way ANOVA was applied to contrast the expression levels of m6A RNA methylation regulators in primary CMM and metastatic CMM groups (TCGA datasets), and *t*-tests were applied to contrast the expression levels in OC patients for grade, age, and survival status. Patients were grouped into two clusters by consensus expression of m6A RNA methylation regulators or were separated into the low-risk and high-risk groups applying the median risk score (came from the risk signature) as the cut-off value. Chi-square tests were applied to contrast the distribution of patients' age, survival status, and the grade between the two risk groups. Univariate and multivariate Cox regression analyses were conducted to evaluate the prognostic value of the risk score and various clinical and molecular-pathological features. The prediction efficiency of the risk signature was tested with the ROC curve. The Kaplan-Meier method with a two-sided log-rank test was applied to contrast the OBS of the patients in the cluster 1/2 groups or in the low- and high-risk groups. All statistical analyses were executed utilizing R v3.4.1 (https://www.r-project.org/) and Prism 8 (GraphPad Software Inc., La Jolla, CA).

## 3. Results and Discussion

### 3.1. M6A Gene-Based Clustering Showed 2 Subtypes of CMM

**Figure 1:**
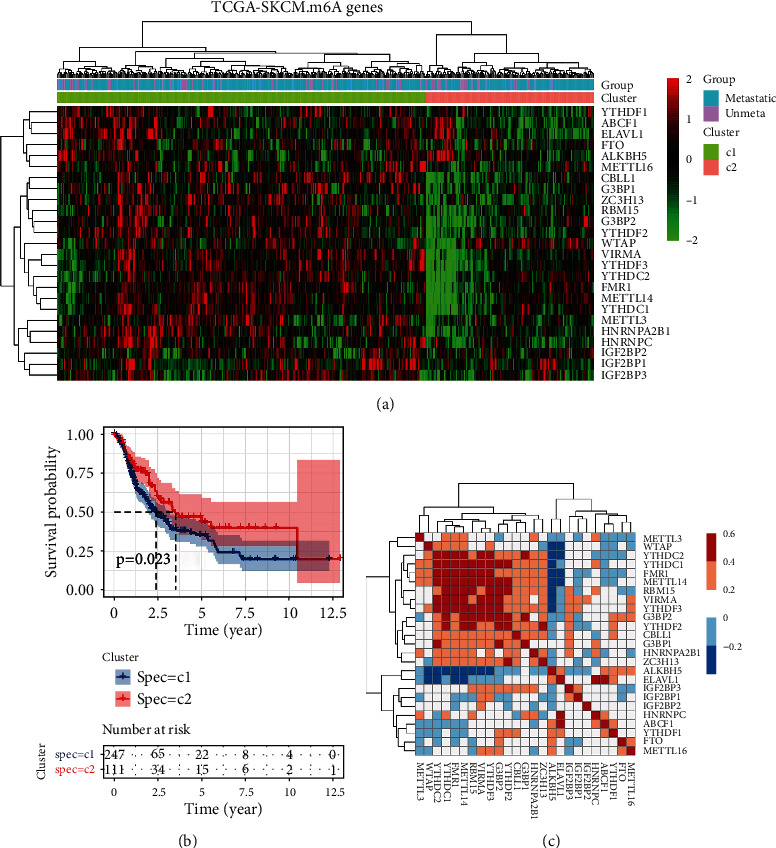
Consensus clustering of m6A RNA methylation regulators identified two clusters of cutaneous malignant melanoma. (a) Heatmap and metastatic status of the two clusters defined by m6A regulators consensus expression. (b) Kaplan-Meier Observed survival interval (OBS) in patients of the two defined cluster. (c) Spearman's correlation analysis of the 25 m6A regulators.

According to the expression profiles of 25 m6A genes, cluster analysis was performed to analyze the 470 CMM samples from the TCGA database (Ward's method). The clustering results showed that two clusters were determined in the CMM samples (Figure 1(a)). The gross m6A gene expression in cluster 2 was lower than that that in cluster 1. We compared OBS curves of the two clusters and found that the survival of cluster 1 was worse than that of cluster 2 (*p* = 0.023), indicating that the expression of m6A genes was negatively correlated with patient prognosis (Figure 1(b)). We further analyzed the correlation between these m6A genes, and there was a strong correlation between YTHDC2, YTHDC1, FMR1, METTL14, RBM15, VIRMA, YTHDF3, and G3BP2 (Figure 1(c)). It is known that metastasis is an important factor affecting the survival of malignant melanoma patients. So we made statistics on the metastasis cases in the two clusters: there are 70 nonmetastatic cases and 253 metastatic cases in cluster 1 and 33 nonmetastatic samples, and 114 metastatic cases in cluster 2 (Figure 1(a)). The chi-square test showed that there is no significant difference in metastatic proportions between these two clusters (*p* = 0.852), indicating that the differential expression of m6A genes may not be correlated with the metastasis status of CMM.

### 3.2. Correlation between m6A Genes and Metastasis of CMM

**Figure 2:**
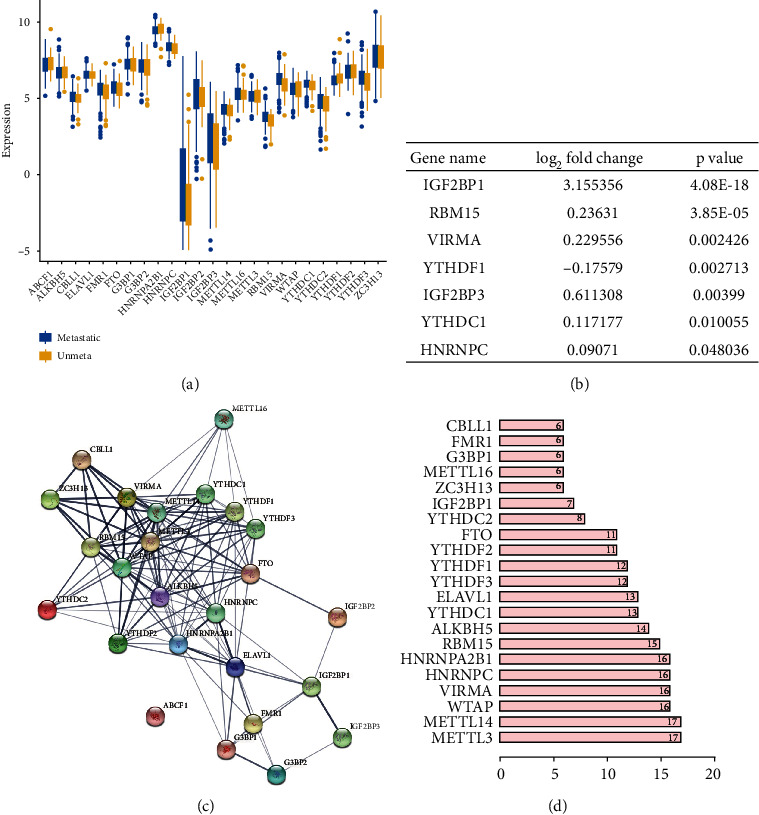
Expression of the m6A RNA methylation regulators and interaction among them. (a) The expression levels of 25 m6A RNA methylation regulators in metastatic CMM (*n* = 367) and nonmetastatic CMM (*n* = 103). (b) Table of the 7 different expressed m6A regulators (*p* < 0.05) between the metastatic CMM group and the nonmetastatic group. (c) The m6A modification-related interactions among the 25 m6A RNA methylation regulators. (d) Number of related nodes of m6A RNA methylation regulators (only showing the number > 5).

Metastasis status is closely related to prognosis in CMM. In the previous part, we found that in the two clusters determined by m6A gene expression, there is no significant difference in metastatic rate. Here, we continued to study whether the m6A genes correlated with the metastasis of CMM. Firstly, we screened the differentially expressed genes from the transcriptome sequencing data of primary and metastatic cases in TCGA CMM database. The *p*-value was calculated by R deseq2 and edge package, respectively. The difference screening standard was *p* < 0.05, and fold change was more than 2 times. Either of the methods calculated that*p* < 0.05is considered to be significantly. Among them, we selectively observed the m6A genes (Figure 2(a)). There were 7 differentially expressed m6A genes (*p* < 0.05) between the primary group and the metastasis group, which were IGF2BP1, RBM15, VIRMA, YTHDF1, IGF2BP3, YTHDC1, and HNRNPC, respectively (Figure 2(b)). The expression of YTHDF1 was downregulated, while that of others was upregulated. The expression change of IGF2BP1 was the most significant, and its fold change was 8.91, while the other fold changes were less than 2. This confirms what was stated in the previous section that most of the m6A gene expression has little association with metastasis status in CMM except IGF2BP1. We further analyzed the interactions among the 25 m6A regulators. Protein-protein interaction network analysis showed that the writers METTL3, METTL14, WTAP, VIRMA, and RMB15; the erasers ALKBH5 and FTO; and the readers HNRNPC, HNRNPA2B1, ELAVL1, YTHDC1, YTHDF1, YTHDF2, and YTHDF3 had more interactions with each other, while ABCF1, IGF2BP1, IGF2BP2, IGF2BP3, G3BP2, G3BP1, FMR1, and METTL16 had fewer interactions with other regulators (Figures 2(c) and 2(d)). In addition, we further analyzed the interactions between the 25 m6A regulators and other proteins (Figure S1). It is obvious that ELAVL1 interacts most with other proteins, mainly with RNA binding proteins. Our conclusion is that most (but not all) of the 25 m6A regulators are closely related to each other. Among them, ELAVL1 interacts most with other proteins, mainly with RNA binding proteins.

### 3.3. Development of a Risk Signature Consisting of Three m6A RNA Methylation Regulators

**Figure 3:**
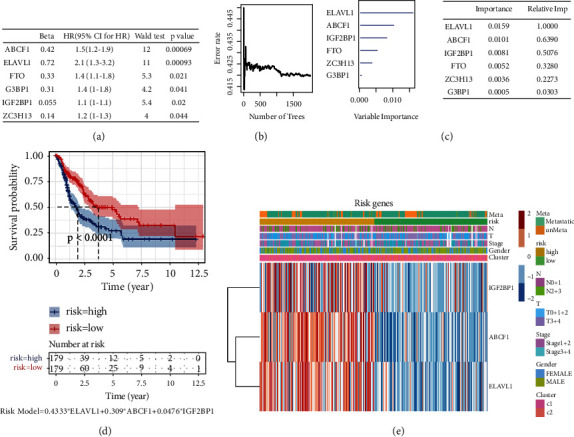
The selection of three m6A RNA methylation regulators and their effect on CMM prognosis and clinicopathological characteristics. (a) Cox univariate regression analyses were used to examine the associations between expression of 25 m6A RNA methylation regulators and prognosis, and six m6A regulators closely related to survival were screened out (*p* < 0.05). (b, c) A random survival forest algorithm was used to analyze the importance of the six regulators, and the relative importance of these regulators is shown. (d) The risk formula was constructed; then the survival analysis was done in the TCGA CMM dataset of the high-risk group and the low-risk group. (e) Heatmap and clinicopathologic features of the three selected m6A RNA methylation regulators. N stands for N classification in TNM system, and T stands for T classification in TNM system.

In order to better predict the clinical outcomes by abnormal expression of m6A genes, we did univariate Cox proportional hazard region analysis to the 25 m6A regulators in the TCGA dataset (Table S2), and six m6A genes closely related to survival were screened out (*p* < 0.05). The six genes were ABCF1, ELAVL1, FTO, G3BP1, IGF2BP1, and ZC3H13 (Figure 3(a)). The importance of the 6 genes was analyzed by random survival forest algorithm; the relative importance of these genes is shown (Figures 3(b) and 3(c)). With the median relative importance (median relative importance = 0.5) as the cut-off point, ELAVL1, ABCF1, and IGF2BP1 were selected as the most important m6A genes. Therefore, the three regulators were used for multivariate regression analysis, and the risk formula was constructed according to the Cox regression results (Table S3). The risk score formula was as follows: risk score = 0.4333∗ELAVL1 + 0.309∗ABCF1 + 0.0476∗IGF2BP1. The risk score of each patient in TCGA dataset was calculated according to the formula, and patients were classified into two groups according to the median risk score: high-risk group and low-risk group. Then, the survival analysis was performed in the high-risk group and the low-risk group; the results showed that the survival of the high-risk group was significantly worse than that of the low-risk group (*p* < 0.0001) (Figure 3(d)), which were similar to the result of total m6A genes clustering but with a smaller *p* value. Therefore, through importance analysis, this study successfully constructed a risk formula with three m6A genes as variables, which can accurately predict the prognosis of CMM patients. In addition, we compared the clinicopathological characters (including gender, age, stage, T, N, and risk score) of the three m6A genes. The results shown that the expressions of the three selected m6A genes were all high in most high-risk patients (Figure 3(e)). In addition, univariate and multivariate analyses were performed to assess whether clinicopathological features were independent prognostic factors. Univariate analysis of all variables using a Cox proportional hazard model showed that risk status (*p* = 0.002, 95% CI HR 0.38-0.7) and primary tumor status (*p* = 0.016, 95% CI HR 1.1-2.1) were independent prognostic factors. Multivariate analysis used the same variables as univariate analysis in the cohort and supported that risk status (*p* < 0.001, 95% CI HR 0.357-0.691) and primary tumor status (*p* = 0.0108, 95% CI HR 1.11-2.21) were independent prognostic factors. Little association of survival with tumor stages was discovered, so we analyzed the expression profiles of ELAVL1, ABCF1, and IGF2BP1 in CMM patients with different tumor stages in TCGA database on the UALCAN website (http://ualcan.path.uab.edu); the results demonstrated that all these 3 genes have no significant correlation with tumor stage in cutaneous melanoma (Figure S2).

**Figure 4:**
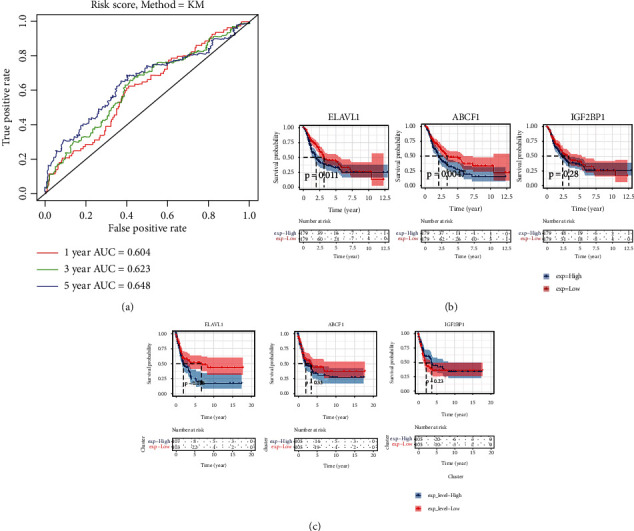
The prognosis analysis and validation in the GEO database of the three selected m6A RNA methylation regulators. (a) The ROC curve showed the predictive efficiency of the risk signature on CMM. (b) OBS survival curve of CMM patients based on the three selected m6A RNA methylation regulators levels in the TCGA dataset. (c) The validation of prognosis efficiency of the three selected m6A RNA methylation regulators using the GEO database (GSE65904).

The receiver operating characteristic (ROC) analysis was used to testing if the survival prediction based on the risk score is sensitive and specific. The calculation of the area under the curve (AUC) values was carried out according to ROC curves. The ROC curves of 1 year, 3 years, and 5 years are made, respectively, the AUC of 1 year is 0.604, the AUC of 2 years is 0.623, and the AUC of 3 years is 0.648, which are all greater than 0.6 (Figure 4(a)). We further explored the prognostic importance of each individual m6A regulators of the signature composed of ELAVL1, ABCF1, and IGF2BP1; the survival course of patients with a high expression level of any gene of the signature was compared to that of patients with low expression. Through the TCGA CMM dataset, we found that high ELAVL1 (*p* = 0.011) and ABCF1 (*p* = 0.0047) expression are negative correlate with patient survival. However, IGF2BP1 expression does not associate with the OBS of CMM patients (Figure 4(b)).

Finally, we validated the risk signature developed in this study using GEO dataset (GSE65904). Univariate Cox proportional hazard region analysis to the 25 m6A regulators in the GEO dataset showed that ELAVL1 owned the smallest *p* value (Table S4), which proved the important role of ELAVL1 in survival prediction. Similar with the TCGA dataset, the survival of the high-risk group determined by above three m6A genes was significantly worse than that of the low-risk group (*p* = 0.011) (Figure S3). This result further confirmed the efficiency of the risk signature to predict prognosis in CMM patients. The ROC curves of 1 year, 3 years, and 5 years were also made, respectively, the AUC of 1 year is 0.57, the AUC of 2 years is 0.64, and the AUC of 3 years is 0.712 (Figure S4). However, individual prognostic effect analysis showed that only ELAVL1 expression of the three m6A genes was significantly correlated (*p* = 0.018) with survival of GSE65904 data (Figure 4(c)).

### 3.4. Gene Set Enrichment Analysis (GSEA)

**Figure 5:**
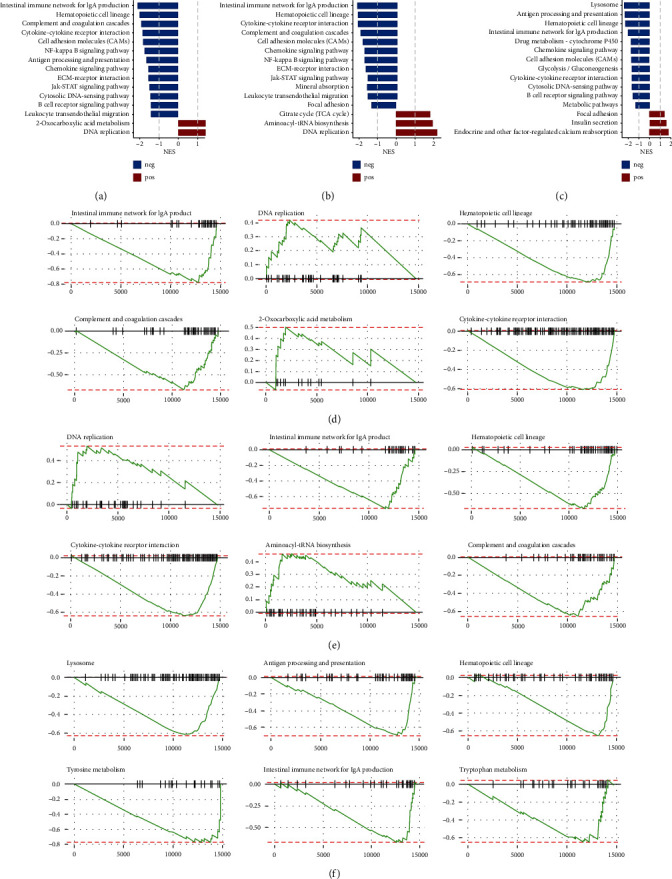
Gene set enrichment analysis of the three selected m6A regulators. (a) The top 15 nondisease-related pathways in the ELAVL1 high expression group. (b) The top 15 nondisease-related pathways in the ABCF1 high expression group. (c) The top 15 nondisease-related pathways in the IGF2BP1 high expression group. (d) Enriched gene sets in the first 6 enriched pathways in the ELAVL1 high expression group. (e) Enriched gene sets in the first 6 enriched pathways in the ABCF1 high expression group. (f) Enriched gene sets in the first 6 enriched pathways in the IGF2BP1 high expression group.

The GSEA of the three selected genes were analyzed, and the possible biological functions of the three genes were speculated. The samples were divided into the high expression group and the low expression group according to the median value of the gene expression. The LFCs of all genes detected in TCGA data of high expression group and low expression group were calculated and used for GSEA analysis. The path with adjusted *p* < 0.05 and absolute value of NES ≥ 1 in the screening results is considered to be the enriched pathway. The top 15 nondisease-related pathways were selected according to the absolute value of NES. In the high expression groups of ELAVL1, ABCF1, and IGF2BP1, we noticed that the several inflammatory and immune related signaling pathways were negatively correlated (Figures 5(a)–5(c)). Involved biological processes including intestine immune network for IgA production, NF-kappaB signaling, cytokine-cytokine receptor pathway, and antigen processing and presentation. We further examine the enriched gene sets in the first 6 enriched pathway in samples with high ELAVL1, ABCF1, and IGF2BP1 mRNA expression levels, and the results indicated that the gene sets of intestine immune network for IgA production were all negative correlated with these three gene expressions (Figures 5(d)–5(f)). This suggests that these three genes may participate in tumor immunity and affect tumor prognosis. However, the related mechanisms need to be further explored.

### 3.5. Analysis of m6A Gene Mutation and Prognosis

**Figure 6:**
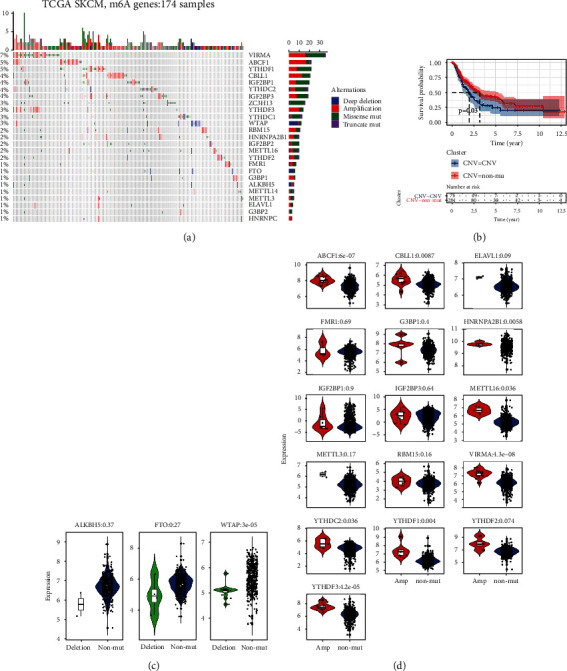
Analysis of m6A gene mutation and prognosis. (a) The landscape of mutation information in m6A genes in TCGA dataset. The top panel showed the number of mutations in each sample, and the bottle panel exhibited the mutation types of high-frequency mutated genes with various colors in each sample. (b) Survival curve of the CNV-mutation group and non-CNV mutation group. (c) The expression of ALKBH5, FTO, and WTAP was downregulated in the mutant cases. (d) The expression of other m6A regulators was upregulated in the mutant cases.

We further analyzed the relationship between m6A gene mutation and patient prognosis of CMM. A total of 174 patients' mutation information was download in this dataset (Figure 6(a)). The patients were divided into two groups according to whether there was any mutation in the m6A genes. The univariate Cox regression analysis was performed to detect whether SNV, CNV, SNV, or CNV in the m6A gene is correlated with prognosis. The results demonstrated that CNV is significantly correlated to the prognosis (*p* = 0.03) (Table S5), and the survival analysis demonstrates that patients with non-CNV mutation in m6A genes is better than that of patients with CNV mutation (Figure 6(b)). We further analyzed whether CNV mutation affected prognosis by affecting m6A gene expression. We found that the expression of ALKBH5, FTO, and WTAP was downregulated in the mutant cases (Figure 6(c)), and as expected, copy number loss caused by deletion mutation was associated with lower expression of these genes. The expressions of other m6A genes were upregulated (Figure 6(d)), and amplification of copy number was the main reason. Interestingly, two of the three mutant downregulated m6A regulators were erasers. These results suggest that the mutation may upregulate the methylation of m6A by reducing the expression of eraser or increasing the expression of other m6A regulators, which may lead to poor prognosis.

### 3.6. Correlation Analysis of m6A Regulators and Immune Cell Infiltration

**Figure 7:**
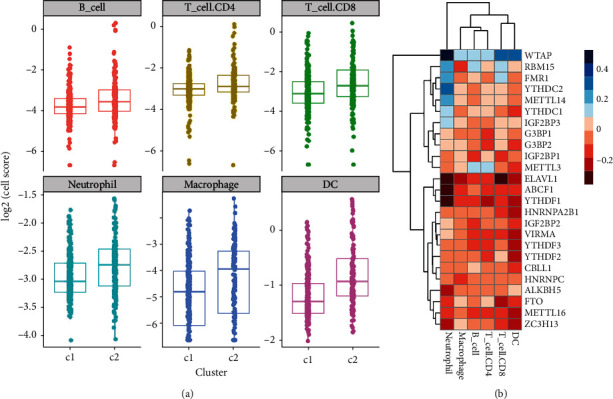
Correlation analysis of m6A regulators and immune cell infiltration. (a) The infiltration of B lymphocytes, CD4 T lymphocytes, CD8 T lymphocytes, neutrophils, macrophages, and dendritic cells in cluster 1 and cluster 2 was analyzed by TME. (b) The correlation of immune cell infiltration and individual m6A regulator expression.

The tumor microenvironment plays an important role in carcinogenesis, especially in therapeutic efficacy of immunotherapy. Large numbers of immune cells could infiltrate in the microenvironment. These cells exert various functions, ranging from regulating the immune process to drug sensitivity. Previous GSEA analysis showed that inflammatory and immune-related signaling pathways were downregulated in ELEVL1, ABCF1, and IGF2BP1 high expression cases, which indicated that they may participant in tumor immunity regulation. Therefore, we examined whether the expression of m6A genes is correlated with immune cell infiltration in CMM. The proportion of B lymphocytes, CD4 T lymphocytes, CD8 T lymphocytes, neutrophils, macrophages, and dendritic cells in cluster 1 and cluster 2 was analyzed by TME. It was found that all the 6 types of cells were decreased in cluster 1 (Figure 7(a)). The results demonstrated that high gross m6A gene expression may negative correlated with immune cell infiltration. We further analyzed the correlation of immune cell infiltration and individual m6A gene expression. The result showed that generally the m6A gene expressions are negative correlated with all 6 immune cell type infiltration expect WTAP. And among these m6A genes, ELAVL1, YTHDF1, and ABCF1 are more interaction with immune cell infiltration, and neutrophils are the most interaction cell type (Figure 7(b)). Thus, two of the three risk signature genes found in this study were closely and negatively related with immune cell infiltration, which may partly explain the prognosis mechanism of the risk signature.

## 4. Discussion

Cutaneous melanoma is one of the most common malignant tumors of the skin, which seriously threatens public health. The development of CMM is closely associated with epigenetic modification, such as DNA methylation, histone modification, and RNA editing [25–28]. m6A is a common internal trim of RNA molecules. The modification level of transcript m6A is dynamically regulated by methyltransferase (writer), binding protein (reader), and demethylase (erase). The discovery of m6A RNA methylation regulators has greatly improved our understanding of the functions and mechanisms of m6A modification in the posttranscriptional gene regulation [14, 29].

m6A methylation regulators may have similar effects on different types of cancers or play different roles in similar types of cancers [30–33]. Although the roles of m6A regulators in cancer have been widely studied, there is neither a comprehensive analysis of the expression of m6A genes in CMM nor a study on their functions in the prognostic values. Here, we firstly analyzed the expression of 25 m6A genes in CMM and the relationship between their expressions. Considering the low proportion of melanocyte accounting for normal skin tissue, it is meaningless to compare malignant melanoma tissue with normal skin tissue. So in this study, we applied the consensus clustering to divide all CMM samples into two clusters and analyzed the expression of m6A genes and different clinicopathological variables between these two clusters. We found that high m6A expression CMM patients had a worse survival.

Next, we explored the prognostic value of each m6A genes and developed a risk signature applying three m6A genes, ELAVL1, ABCF1, and IGF2BP1, which are selected by the univariate Cox analysis and LASSO Cox regression analysis. ELAVL1, also named human antigen R (HuR), is a member of the ELAVL family encoding RNA-binding proteins that contain several RNA recognition motifs and selectively bind AU-rich elements (AREs) found in the 3′ untranslated regions of mRNAs. ARE signal degradation of mRNAs is a means to regulate gene expression; thus by binding AREs, the ELAVL family of proteins plays a role in stabilizing ARE-containing mRNAs. ELAVL1 has been implicated in a variety of biological processes, and it is highly expressed in many cancers and could be potentially useful in cancer diagnosis, prognosis, and therapy [34–37]. Ahmed et al. reported that anti-HuR could suppress MITF expression and induces apoptosis in melanoma cells, which shows potential in melanoma therapy. IGF2BP1 (insulin-like growth factor 2 mRNA binding protein 1) plays significant roles in carcinogenesis, including tumor cell proliferation and growth, invasion, and chemoresistance, and is associated with poor overall survival and metastasis in various types of human cancers [38, 39]. It is reported that IGF2BP1 accelerated melanoma cell metastasis and target inhibition enhances the effects of BRAF-inhibitor and BRAF-MEK inhibitors in BRAF mutation melanoma [40, 41]. Interestingly, IGF2BP1 is the most upregulated m6A gene in metastatic samples found in this study. However, whether IGF2BP1 promotes melanoma metastasis partly by regulation m6A methylation needs further study. The protein encoded by ABCF1 is a member of the superfamily of ATP-binding cassette (ABC) transporters. This protein is a member of the GCN20 subfamily. Unlike other members of the superfamily, this protein lacks the transmembrane domains which are characteristics of most ABC transporters. This protein may be a hepatic oncofetal protein that promotes chemoresistance, EMT, and cancer stemness in hepatocellular carcinoma [42]. ABCF1 also plays an important role in the chemoresistance of colorectal cancer cells with microsatellite instability to 5-fluorouracil [43]. The role of ABCF1 in melanoma has not been reported, but in our PPI analysis, we found that ABCF1 was indirectly correlated with ELAVL1, which may be the mechanism how it participates in melanoma progress. However, more mechanisms need to be further explored. Based on this signature, we established a nomogram that assimilated the three selected m6A genes associated with CMM prognosis and used univariate analysis and multivariate analysis to assess the prognostic value of the three m6A genes. At last, the prognostic value of the three selected m6A genes was validated by a different CMM cohort. The result showed that these risk signatures correlated well with patient prognosis. This work provided a different biomarker other than the tumor stage for predicting the prognosis of CMM.

Gene mutation, including copy number variation (CNVs) and single-nucleotide polymorphism (SNPs), plays important roles in the development and progression of human cancers [44, 45]. However, the effect of CNV and SNP in m6A-related genes remain unknown to us. In this study, we comprehensively explored the effect of CNVs and SNP mutations in m6A genes on the mRNA expression and patient prognosis. We observed that CNVs affected patient prognosis; thus, the survival of CNVs patients was worse than that of non-CNV mutation patients. Furthermore, we observed that whether CNV mutation dysregulated mRNA expression to correlate with patient prognosis. Our data shows that the expression of ALKBH5, FTO, and WTAP was downregulated in the mutation group, which was due to the deletion mutation. And the other m6A genes' expression was upregulated in the mutation group. Interestingly, the 2 of the 3 downregulated m6A genes were the “erase,” thus ALKBH5 and FTO. Thus, the mutation in m6A genes grossly upregulated the expression of m6A methylation level, so as to harbor a worse prognosis. This also suggests that normal m6A process plays an important role in the development of CMM.

In the selected three risk signature m6A regulators, GSEA analysis indicated that they were closely correlated with inflammation and immune pathways. Cancer cells elicited multiple biological behavior changes through direct and indirect interactions with other TME components such as inducing proliferation and angiogenesis, inhibiting apoptosis, avoiding hypoxia, and inducing immune tolerance. As the understanding of the diversity and complexity of tumor microenvironment has deepened, emerging evidence reveals its critical role in the tumor progression, immune escape, and its effect on response to immunotherapy. Predicting the response to ICB based on the characterization of TME cell infiltration is a key procedure on increasing the success of existing ICBs and exploiting novel immunotherapeutic strategies [46, 47]. We further analyze whether m6A regulators affect patient prognosis by affecting immune cell infiltration in tumor microenvironment. Increasing evidence demonstrated that m6A modification took on an indispensable role in inflammation, innate immunity, and antitumor effect through interaction with various m6A regulators. As most studies focus on single TME cell type or single regulator, the overall TME infiltration characterizations mediated by integrated roles of multiple m6A regulators are not comprehensively recognized. Identifying the role of distinct m6A modification patterns in the TME cell infiltration will contribute to enhancing our understanding of TME antitumor immune response and guiding more effective immunotherapy strategies. Based on 25 m6A genes, we divided the TCGA dataset into 2 clusters, and we further analyze the immune cell infiltration in these 2 clusters. The results demonstrated that all the 6 cell types: B cells, CD4+T cells, CD8+T cells, neutrophils, macrophages, and DCs, are downregulated in cluster 1, as the gross m6A genes are highly expressed in cluster 1; we guess that the m6A regulators may negatively correlate with immune cell infiltration. Then, we further analyzed the correlation of every single m6A gene and immune cell infiltration. We found that generally the m6A genes are negatively correlated with all 6 immune cell types expect WTAP. And among these genes, ELAVL1, YTHDF1, and ABCF1 are more interaction with immune cell infiltration, and neutrophils are the most involved cell type. Interestingly, ELAVL1 and ABCF1 are the risk signature regulators found in this study. We speculate that the risk signatures may be correlated to the prognosis of CMM patients partly by affecting the local immune microenvironment.

## 5. Conclusion

In conclusion, our studies comprehensively manifested the expression and prognostic value of m6A genes in CMM and derived a risk signature consisting of three selected m6A genes as an independent prognostic marker for CMM. These three m6A gene expressions are also negatively correlated with immune cell infiltration in tumor microenvironment, which partly explains the prognostic mechanism of the m6A signature. In brief, our study provides novel markers for evaluating CMM prognosis and furnishes significant proof for future research on the role of RNA m6A methylation in CMM.

## Data Availability

Publicly available datasets were analyzed in this study. This data can be found here: https://www.ncbi.nlm.nih.gov/geo/ and https://portal.gdc.cancer.gov/.

## Supplementary Materials

Supplementary Materials Table S1: list of 25 m6A genes. Table S2: univariate Cox proportional hazard region analysis to the 25 m6A genes in the TCGA dataset. Table S3: Cox regression results of selected m6A genes. Table S4: univariate Cox proportional hazard region analysis to the 25 m6A regulators in the GEO dataset (GSE65904). Table S5: Cox regression analysis of CNV, SNV, CNV, or SNV in m6A genes with prognosis. Figure S1: the interactions between the 25 m6A RNA methylation regulators and other proteins. ELAVL1 interacts most with other proteins, mainly with RNA binding proteins. Figure S2: the expression profiles of ELAVL1, ABCF1 and IGF2BP1 in CMM patients with different tumor stages. All these 3 genes have no significant correlation with tumor stage in cutaneous melanoma. Figure S3: the risk formula was constructed; then the survival analysis was done in the GSE65904 dataset of the high-risk group and the low-risk group. Similar with the TCGA dataset, the survival of the high-risk group was significantly worse than that of the low-risk group (*p* = 0.011). Figure S4: the ROC curve showed the predictive efficiency of the risk signature on GSE65904 dataset. The AUC of 1 year is 0.57, the AUC of 2 years is 0.64, and the AUC of 3 years is 0.712.
