# Supplementary material for: M6A-Related Bioinformatics Analysis Reveals a New Prognostic Risk Signature in Cutaneous Malignant Melanoma
Source: Dis Markers. 2022 Jun 6;2022:8114731. doi: 10.1155/2022/8114731 (PMC9201746; doi:10.1155/2022/8114731)
Supplement: Supplementary Materials — Table S1: list of 25 m6A genes. Table S2: univariate Cox proportional hazard region analysis to the 25 m6A genes in the TCGA dataset. Table S3: Cox regression results of selected m6A genes. Table S4: univariate Cox proportional hazard region analysis to the 25 m6A regulators in the GEO dataset (GSE65904). Table S5: Cox regression analysis of CNV, SNV, CNV, or SNV in m6A genes with prognosis. Figure S1: the interactions between the 25 m6A RNA methylation regulators and other proteins. ELAVL1 interacts most with other proteins, mainly with RNA binding proteins. Figure S2: the expression profiles of ELAVL1, ABCF1 and IGF2BP1 in CMM patients with different tumor stages. All these 3 genes have no significant correlation with tumor stage in cutaneous melanoma. Figure S3: the risk formula was constructed; then the survival analysis was done in the GSE65904 dataset of the high-risk group and the low-risk group. Similar with the TCGA dataset, the survival of the high-risk group was significantly worse than that of the low-risk group (p = 0.011). Figure S4: the ROC curve showed the predictive efficiency of the risk signature on GSE65904 dataset. The AUC of 1 year is 0.57, the AUC of 2 years is 0.64, and the AUC of 3 years is 0.712. [file 8114731.f1.zip › Supplementary Table-revised (1).docx]

Table S1: List of 25 m6A genes.

| **Category** | **m6A regulator** | **Reference** |
| --- | --- | --- |
| **Writer** | METTL3 | Liu J, et al.^1^, Vu LP, et al.^2^, |
|  | METTL14 | Liu J, et al.^1^, Wang X, et al^3^, |
|  | METTL16 | Su R, et al.^4^, Aoyama T, et al^5^, |
|  | WTAP | Ping XL, et al.^6^ |
|  | VIRMA | Yue Y, et al.^7^ |
|  | RMB15 | Xie Y, et al.^8^ |
|  | ZC3H13 | Knuckles P, et al.^9^ |
|  | CBLL1 | Zhang X, et al.^10^ |
| **Eraser** | FTO | Jia G, et al.^11^, Niu Y, et al.^12^ |
|  | ALKBH5 | Zheng G, et al.^13^, Yu H, et al.^14^ |
| **Reader** | YTHDF1 | Liu T, et al.^15^, Zhang Z, et al.^16^ |
|  | YTHDF2 | Du H, et al.^17^ |
|  | YTHDF3 | Shi H, et al.^18^ |
|  | YTHDC1 | Xu C, et al.^19^ Xiao W, et al.^20^ |
|  | YTHDC2 | Hsu PJ, et al.^21^ |
|  | IGF2BP1 | Muller S, et al.^22^, Zhu S, et al.^23^ |
|  | IGF2BP2 | Hu X, et al.^24^ |
|  | IGF2BP3 | Yang Z, et al.^25^ |
|  | HNRNPA2B1 | Alarcón CR, et al.^26^ |
|  | HNRNPC | Wang LC, et al.^27^ |
|  | G3BP1 | Matsuki H, et al.^28^, Edupuganti, RR, et al.^29^ |
|  | G3BP2 | Matsuki H, et al.^28^, |
|  | ABCF1 | Coots RA, et al.^30^ |
|  | ELAVL1 | Ling Z, et al.^31^ |
|  | FMR1 | Ascano M, et al.^32^ |

**Reference**

1. Liu J, Yue Y, Han D, Wang X, Fu Y, Zhang L, et al. A METTL3-METTL14 complex mediates mammalian nuclear RNA N6-adenosine methylation. Nat Chem Biol. 2014 Feb;10(2):93-5.

2. Vu LP, Pickering BF, Cheng Y, Zaccara S, Nguyen D, Minuesa G, et al. The N6-methyladenosine (m6A)-forming enzyme METTL3 controls myeloid differentiation of normal hematopoietic and leukemia cells. Nat Med. 2017 Nov;23(11):1369-1376.

3. Wang X, Feng J, Xue Y, Guan Z, Zhang D, Liu Z, et al. Structural basis of N(6)-adenosine methylation by the METTL3-METTL14 complex. Nature. 2016 Jun 23;534(7608):575-8.

4. Su R, Dong L, Li Y, Gao M, He PC, Liu W, et al. METTL16 exerts an m6A-independent function to facilitate translation and tumorigenesis. Nat Cell Biol. 2022 Feb;24(2):205-216.

5. Aoyama T, Yamashita S, Tomita K. Mechanistic insights into m6A modification of U6 snRNA by human METTL16. Nucleic Acids Res. 2020 May 21;48(9):5157-5168.

6. Ping XL, Sun BF, Wang L, Xiao W, Yang X, Wang WJ, et al. Mammalian WTAP is a regulatory subunit of the RNA N6-methyladenosine methyltransferase. Cell Res. 2014 Feb;24(2):177-89.

7. Yue Y, Liu J, Cui X, Cao J, Luo G, Zhang Z, et al. VIRMA mediates preferential m6A mRNA methylation in 3'UTR and near stop codon and associates with alternative polyadenylation. Cell Discov. 2018 Feb 27;4:10.

8. Xie Y, Castro-Hernández R, Sokpor G, Pham L, Narayanan R, Rosenbusch J, et al. RBM15 Modulates the Function of Chromatin Remodeling Factor BAF155 Through RNA Methylation in Developing Cortex. Mol Neurobiol. 2019 Nov;56(11):7305-7320.

9. Knuckles P, Lence T, Haussmann IU, Jacob D, Kreim N, Carl SH, et al. Zc3h13/Flacc is required for adenosine methylation by bridging the mRNA-binding factor Rbm15/Spenito to the m6A machinery component Wtap/Fl(2)d. Genes Dev. 2018 Mar 1;32(5-6):415-429.

10. Zhang X, Zhang S, Yan X, Shan Y, Liu L, Zhou J, et al. m6A regulator-mediated RNA methylation modification patterns are involved in immune microenvironment regulation of periodontitis. J Cell Mol Med. 2021 Apr;25(7):3634-3645.

11. Jia G, Fu Y, Zhao X, Dai Q, Zheng G, Yang Y, et al. N6-methyladenosine in nuclear RNA is a major substrate of the obesity-associated FTO. Nat Chem Biol. 2011 Oct 16;7(12):885-7.

12. Niu Y, Zhao X, Wu YS, Li MM, Wang XJ, Yang YG. N6-methyl-adenosine (m6A) in RNA: an old modification with a novel epigenetic function. Genomics Proteomics Bioinformatics. 2013 Feb;11(1):8-17.

13. Zheng G, Dahl JA, Niu Y, Fedorcsak P, Huang CM, Li CJ, et al. ALKBH5 is a mammalian RNA demethylase that impacts RNA metabolism and mouse fertility. Mol Cell. 2013 Jan 10;49(1):18-29.

14. Yu H, Yang X, Tang J, Si S, Zhou Z, Lu J, et al. ALKBH5 Inhibited Cell Proliferation and Sensitized Bladder Cancer Cells to Cisplatin by m6A-CK2α-Mediated Glycolysis. Mol Ther Nucleic Acids. 2020 Oct 22;23:27-41.

15. Liu T, Wei Q, Jin J, Luo Q, Liu Y, Yang Y, et al. The m6A reader YTHDF1 promotes ovarian cancer progression via augmenting EIF3C translation. Nucleic Acids Res. 2020 Apr 17;48(7):3816-3831.

16. Zhang Z, Theler D, Kaminska KH, Hiller M, de la Grange P, Pudimat R, et al. The YTH domain is a novel RNA binding domain. J Biol Chem. 2010 May 7;285(19):14701-10.

17. Du H, Zhao Y, He J, Zhang Y, Xi H, Liu M, et al. YTHDF2 destabilizes m(6)A-containing RNA through direct recruitment of the CCR4-NOT deadenylase complex. Nat Commun. 2016 Aug 25;7:12626.

18. Shi H, Wang X, Lu Z, Zhao BS, Ma H, Hsu PJ, et al. YTHDF3 facilitates translation and decay of N6-methyladenosine-modified RNA. Cell Res. 2017 Mar;27(3):315-328.

19. Xu C, Wang X, Liu K, Roundtree IA, Tempel W, Li Y, et al. Structural basis for selective binding of m6A RNA by the YTHDC1 YTH domain. Nat Chem Biol. 2014 Nov;10(11):927-9.

20. Xiao W, Adhikari S, Dahal U, Chen YS, Hao YJ, Sun BF, et al. Nuclear m(6)A Reader YTHDC1 Regulates mRNA Splicing. Mol Cell. 2016 Feb 18;61(4):507-519.

21. Hsu PJ, Zhu Y, Ma H, Guo Y, Shi X, Liu Y, et al. Ythdc2 is an N6-methyladenosine binding protein that regulates mammalian spermatogenesis. Cell Res. 2017 Sep;27(9):1115-1127.

22. Müller S, Glaß M, Singh AK, Haase J, Bley N, Fuchs T, et al. IGF2BP1 promotes SRF-dependent transcription in cancer in a m6A- and miRNA-dependent manner. Nucleic Acids Res. 2019 Jan 10;47(1):375-390.

23. Zhu S, Wang JZ, Chen D, He YT, Meng N, Chen M, et al. An oncopeptide regulates m6A recognition by the m6A reader IGF2BP1 and tumorigenesis. Nat Commun. 2020 Apr 3;11(1):1685.

24. Hu X, Peng WX, Zhou H, Jiang J, Zhou X, Huang D, et al. IGF2BP2 regulates DANCR by serving as an N6-methyladenosine reader. Cell Death Differ. 2020 Jun;27(6):1782-1794.

25. Yang Z, Wang T, Wu D, Min Z, Tan J, Yu B. RNA N6-methyladenosine reader IGF2BP3 regulates cell cycle and angiogenesis in colon cancer. J Exp Clin Cancer Res. 2020 Sep 29;39(1):203.

26. Alarcón CR, Goodarzi H, Lee H, Liu X, Tavazoie S, Tavazoie SF. HNRNPA2B1 Is a Mediator of m(6)A-Dependent Nuclear RNA Processing Events. Cell. 2015 Sep 10;162(6):1299-308.

27. Wang LC, Chen SH, Shen XL, Li DC, Liu HY, Ji YL, et al. M6A RNA Methylation Regulator HNRNPC Contributes to Tumorigenesis and Predicts Prognosis in Glioblastoma Multiforme. Front Oncol. 2020 Oct 8;10:536875.

28. Matsuki H, Takahashi M, Higuchi M, Makokha GN, Oie M, Fujii M. Both G3BP1 and G3BP2 contribute to stress granule formation. Genes Cells. 2013 Feb;18(2):135-46.

29. Edupuganti RR, Geiger S, Lindeboom RGH, Shi H, Hsu PJ, Lu Z, et al. N6-methyladenosine (m6A) recruits and repels proteins to regulate mRNA homeostasis. Nat Struct Mol Biol. 2017 Oct;24(10):870-878.

30. Coots RA, Liu XM, Mao Y, Dong L, Zhou J, Wan J, et al. m6A Facilitates eIF4F-Independent mRNA Translation. Mol Cell. 2017 Nov 2;68(3):504-514.e7.

31. Ling Z, Chen L, Zhao J. m6A-dependent up-regulation of DRG1 by METTL3 and ELAVL1 promotes growth, migration, and colony formation in osteosarcoma. Biosci Rep. 2020 Apr 30;40(4):BSR20200282.

32. Ascano M Jr, Mukherjee N, Bandaru P, Miller JB, Nusbaum JD, Corcoran DL, et al. FMRP targets distinct mRNA sequence elements to regulate protein expression. Nature. 2012 Dec 20;492(7429):382-6.

Table S2: Univariate Cox proportional hazards region analysis to the 25 m6A genes in the TCGA dataset

| m6A Gene | Beta | HR (95% CI for HR) for HR) | Wald Test | p Value |
| --- | --- | --- | --- | --- |
| ABCF1 | 0.42 | 1.5 (1.2-1.9).9) | 12 | 0.00069 |
| ALKBH5 | 0.29 | 1.3 (1-1.8)) | 3.8 | 0.051 |
| CBLL1 | 0.046 | 1 (0.77-1.4)4) | 0.09 | 0.77 |
| ELAVL1 | 0.72 | 2.1 (1.3-3.2).2) | 11 | 0.00093 |
| FMR1 | -0.13 | 0.88 (0.71-1.1)-1.1) | 1.6 | 0.21 |
| FTO | 0.33 | 1.4 (1.1-1.8).8) | 5.3 | 0.021 |
| G3BP1 | 0.31 | 1.4 (1-1.8)) | 4.2 | 0.041 |
| G3BP2 | 0.18 | 1.2 (0.96-1.5)1.5) | 2.5 | 0.12 |
| HNRNPA2B1 | 0.098 | 1.1 (0.72-1.7)1.7) | 0.21 | 0.65 |
| HNRNPC | 0.35 | 1.4 (0.94-2.1)2.1) | 2.7 | 0.099 |
| IGF2BP1 | 0.055 | 1.1 (1-1.1)) | 5.4 | 0.02 |
| IGF2BP2 | -0.068 | 0.93 (0.84-1)-1) | 1.7 | 0.19 |
| IGF2BP3 | 0.059 | 1.1 (0.99-1.1)1.1) | 2.6 | 0.11 |
| METTL14 | -0.073 | 0.93 (0.7-1.2)1.2) | 0.26 | 0.61 |
| METTL16 | 0.25 | 1.3 (0.99-1.7)1.7) | 3.7 | 0.056 |
| METTL3 | -0.27 | 0.77 (0.57-1)-1) | 3 | 0.084 |
| RBM15 | 0.051 | 1.1 (0.78-1.4)1.4) | 0.11 | 0.74 |
| VIRMA | 0.14 | 1.1 (0.89-1.5)1.5) | 1.1 | 0.3 |
| WTAP | -0.23 | 0.79 (0.59-1.1)-1.1) | 2.3 | 0.13 |
| YTHDC1 | 0.15 | 1.2 (0.77-1.8)1.8) | 0.52 | 0.47 |
| YTHDC2 | 0.041 | 1 (0.84-1.3)3) | 0.14 | 0.71 |
| YTHDF1 | 0.26 | 1.3 (0.94-1.8)1.8) | 2.5 | 0.11 |
| YTHDF2 | 0.06 | 1.1 (0.81-1.4)1.4) | 0.19 | 0.66 |
| YTHDF3 | 0.17 | 1.2 (0.95-1.5)1.5) | 2.3 | 0.13 |
| ZC3H13 | 0.14 | 1.2 (1-1.3)) | 4 | 0.044 |
|  |  |  |  |  |

Table S3: Cox regression results of selected m6A genes

| ID | Coef | HR | HR 95% L | HR 95% H | p Value |
| --- | --- | --- | --- | --- | --- |
| ELAVL1 | 0.433334317 | 1.542391783 | 0.960301108 | 2.47731924 | 0.073067568 |
| ABCF1 | 0.309440767 | 1.362662855 | 1.05052394 | 1.767546637 | 0.019737341 |
| IGF2BP1 | 0.047618102 | 1.048770056 | 1.001562392 | 1.098202807 | 0.042723802 |

Table S4: Univariate Cox proportional hazards region analysis to the 25 m6A regulators in the GEO dataset (GSE65904)

| m6A Gene | beta | HR (95% CI for HR) for HR) | Wald Test | p Value |
| --- | --- | --- | --- | --- |
| METTL3 | -0.42 | 0.66 (0.4-1.1)1.1) | 2.6 | 0.1 |
| ALKBH5 | 0.11 | 1.1 (0.73-1.7)1.7) | 0.27 | 0.6 |
| YTHDF3 | 0.26 | 1.3 (0.83-2)2) | 1.3 | 0.25 |
| FMR1 | -0.27 | 0.77 (0.052-11)2-11) | 0.04 | 0.85 |
| RBM15 | 0.43 | 1.5 (0.88-2.7)2.7) | 2.3 | 0.13 |
| IGF2BP2 | 0.17 | 1.2 (0.98-1.4)1.4) | 3.1 | 0.077 |
| YTHDC2 | 0.17 | 1.2 (0.6-2.3).3) | 0.23 | 0.63 |
| YTHDF2 | 0.092 | 1.1 (0.68-1.8)1.8) | 0.14 | 0.7 |
| IGF2BP1 | -0.5 | 0.6 (0.27-1.3)1.3) | 1.5 | 0.21 |
| YTHDF1 | 0.45 | 1.6 (0.94-2.6)2.6) | 3 | 0.085 |
| ELAVL1 | 0.52 | 1.7 (1.1-2.5).5) | 6.8 | 0.0091 |
| IGF2BP3 | -0.12 | 0.88 (0.75-1)-1) | 2.1 | 0.15 |
| METTL14 | 0.1 | 1.1 (0.54-2.2)2.2) | 0.08 | 0.78 |
| FTO | 0.26 | 1.3 (0.76-2.2)2.2) | 0.93 | 0.34 |
| HNRNPC | 0.25 | 1.3 (0.67-2.5)2.5) | 0.59 | 0.44 |
| ZC3H13 | 0.11 | 1.1 (0.34-3.7)3.7) | 0.03 | 0.86 |
| WTAP | -0.7 | 0.5 (0.27-0.9)0.9) | 5.4 | 0.02 |
| YTHDC1 | 0.38 | 1.5 (0.79-2.7)2.7) | 1.5 | 0.23 |
| CBLL1 | -0.15 | 0.86 (0.52-1.4)-1.4) | 0.36 | 0.55 |
| G3BP2 | 0.1 | 1.1 (0.71-1.7)1.7) | 0.2 | 0.65 |
| ABCF1 | 0.32 | 1.4 (0.98-2)2) | 3.3 | 0.068 |
| G3BP1 | 0.25 | 1.3 (0.9-1.8).8) | 1.9 | 0.17 |
| HNRNPA2B1 | 0.38 | 1.5 (0.98-2.2)2.2) | 3.4 | 0.065 |
|  |  |  |  |  |

Table S5: Cox regression analysis of CNV, SNV, CNV or SNV in m6A genes with prognosis

|  | Beta | HR (95% CI for HR) | Wald Test | p Value |
| --- | --- | --- | --- | --- |
| CNV | -0.38 | 0.69 (0.49-0.96) | 4.7 | 0.03 |
| SNV | -0.1 | 0.9 (0.63-1.3) | 0.3 | 0.58 |
| CNV or SNV | -0.17 | 0.84 (0.62-1.1) | 1.2 | 0.28 |
