# Supplementary material for: M6A-Related Bioinformatics Analysis Reveals a New Prognostic Risk Signature in Cutaneous Malignant Melanoma
Source: Dis Markers. 2022 Jun 6;2022:8114731. doi: 10.1155/2022/8114731 (PMC9201746; doi:10.1155/2022/8114731)
Supplement: Supplementary Materials — Table S1: list of 25 m6A genes. Table S2: univariate Cox proportional hazard region analysis to the 25 m6A genes in the TCGA dataset. Table S3: Cox regression results of selected m6A genes. Table S4: univariate Cox proportional hazard region analysis to the 25 m6A regulators in the GEO dataset (GSE65904). Table S5: Cox regression analysis of CNV, SNV, CNV, or SNV in m6A genes with prognosis. Figure S1: the interactions between the 25 m6A RNA methylation regulators and other proteins. ELAVL1 interacts most with other proteins, mainly with RNA binding proteins. Figure S2: the expression profiles of ELAVL1, ABCF1 and IGF2BP1 in CMM patients with different tumor stages. All these 3 genes have no significant correlation with tumor stage in cutaneous melanoma. Figure S3: the risk formula was constructed; then the survival analysis was done in the GSE65904 dataset of the high-risk group and the low-risk group. Similar with the TCGA dataset, the survival of the high-risk group was significantly worse than that of the low-risk group (p = 0.011). Figure S4: the ROC curve showed the predictive efficiency of the risk signature on GSE65904 dataset. The AUC of 1 year is 0.57, the AUC of 2 years is 0.64, and the AUC of 3 years is 0.712. [file 8114731.f1.zip › Supplymentry Figure.pdf]

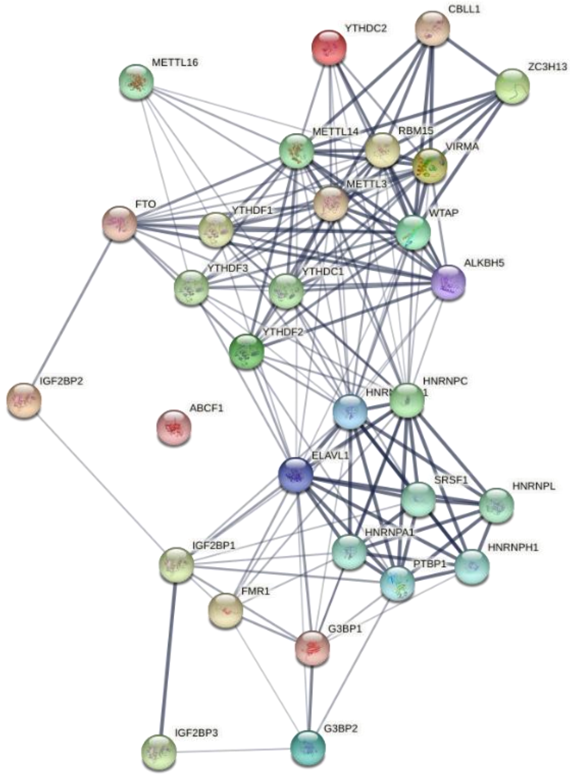

Figure S1: The interactions between the 25 m6A RNA methylation regulators and other proteins. ELAVL1 interacts most with other proteins, mainly with RNA binding proteins.

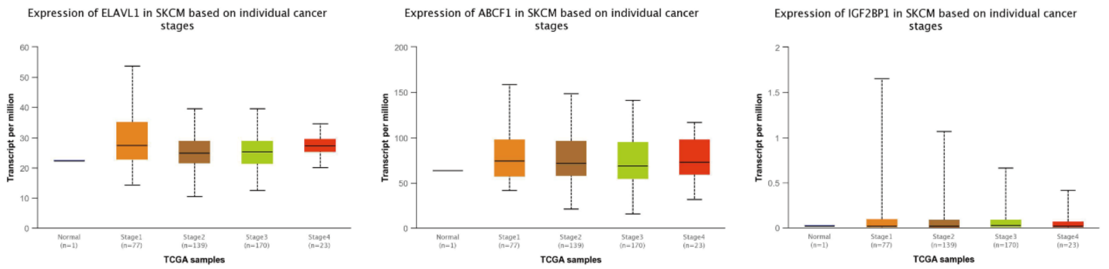

Figure S2: The expression profiles of ELAVL1, ABCF1 and IGF2BP1 in CMM patients with different tumor stages. All these 3 genes have no significant correlation with tumor stage in cutaneous melanoma.

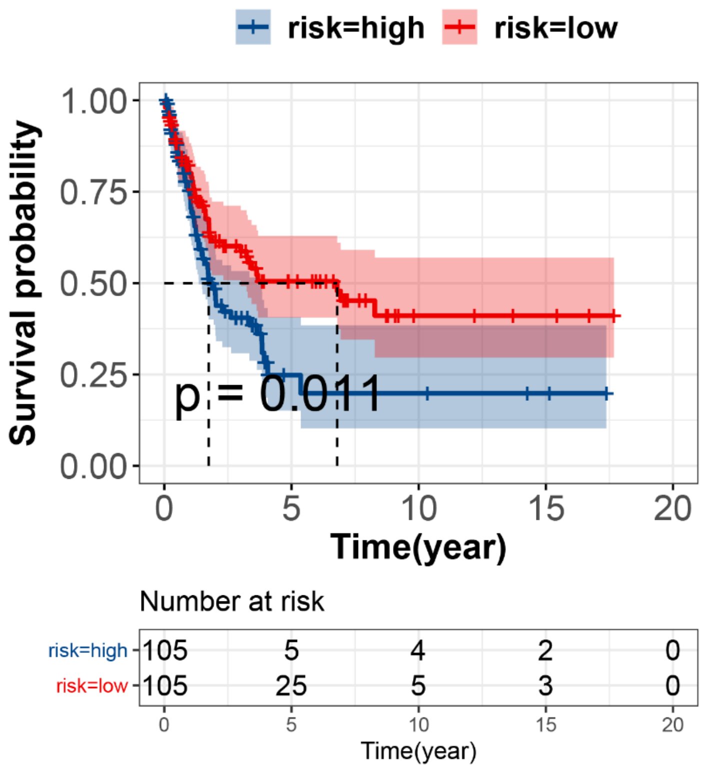

Figure S3: The risk formula was constructed then the survival analysis was done in the GSE65904 dataset of the high-risk group and the low-risk group. Similar with the TCGA dataset, the survival of the high-risk group was significantly worse than that of the low-risk group (p=0.011).

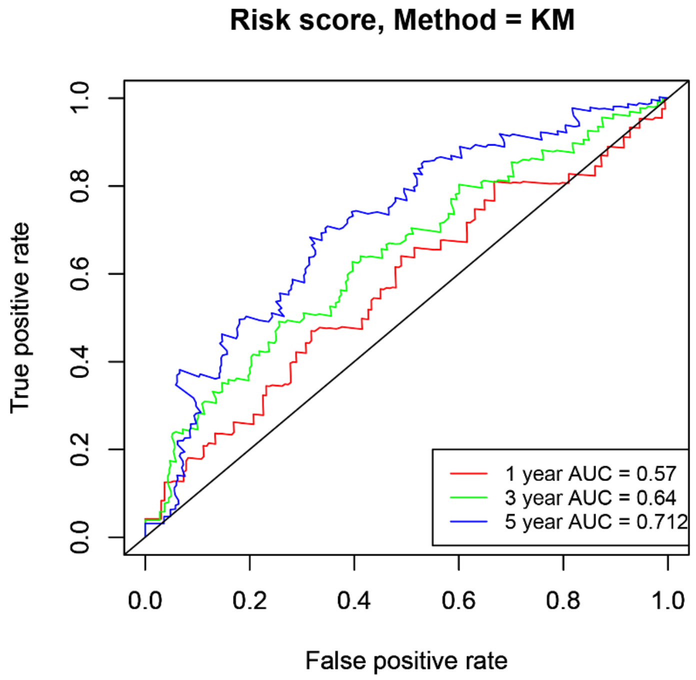

Figure S4: The ROC curve showed the predictive efficiency of the risk signature on GSE65904 dataset. The AUC of 1 year is 0.57, the AUC of 2 years is 0.64, and the AUC of 3 years is 0.712.
